# Six-Month Results of Descemet Membrane Endothelial Keratoplasty in 100 Eyes: First Clinical Results from Turkey

**DOI:** 10.4274/tjo.galenos.2019.27813

**Published:** 2019-10-24

**Authors:** Osman Şevki Arslan, Cezmi Doğan, Burak Mergen

**Affiliations:** 1 İstanbul University-Cerrahpaşa, Cerrahpaşa Faculty of Medicine, Department of Ophthalmology, İstanbul, Turkey

**Keywords:** DMEK, Fuchs’ endothelial dystrophy, pseudophakic bullous keratopathy

## Abstract

**Objectives:**

To evaluate the 6-month outcomes of Descemet membrane endothelial keratoplasty (DMEK) in patients with pseudophakic bullous keratopathy (PBK) and Fuchs’ endothelial dystrophy (FED) in a single center in Turkey.

**Materials and Methods:**

The medical records of patients who underwent DMEK were reviewed retrospectively. Best corrected visual acuity (BCVA), donor corneal endothelial cell density (ECD), donor age, duration in solution after obtaining the donor tissue, and duration after exitus of the donor were evaluated preoperatively and BCVA, ECD, and ECD loss (%) at postoperative 6 months were evaluated postoperatively. Graft detachment, graft failure, and pupillary block were recorded as surgical complications. Patients with cataract and FED underwent combined or staged procedures. Two different graft preparation techniques were utilized: 8 and 9.5 mm.

**Results:**

One hundred eyes of 74 patients were included in the study. Fifty-two of the eyes had FED and the remaining 48 had PBK. Mean ECD loss in 6 months was 29.2±4.4% in the FED group and 29.7±5% in the PBK group (p=0.415). Mean BCVA at 6 months was 0.06±0.05 in the patients with FED and 0.07±0.05 in the patients with PBK (p=0.378). Mean ECD loss in 6 months was 28.3±5.3% in the 8 mm group vs. 29.7±4.5% in the 9.5 mm group (p=0.255), and 28.5±5.6% in the combined group vs. 29.8±2.9% in the staged group (p=0.279).

**Conclusion:**

Different graft preparation techniques can be utilized with similar efficiency for DMEK surgery. A staged or combined approach can be used efficiently in the management of patients with FED and cataract. Our results are promising both for PBK and FED patients.

## Introduction

After the introduction of deep lamellar endothelial keratoplasty (DLEK) in 2001 by Terry and Ousley^1^, a new concept evolved for patients with corneal endothelial pathologies. But the field of keratoplasty took another big step forward with the description of a new technique called Descemet’s stripping endothelial keratoplasty (DSEK) in 2004.^2^ Gorovoy^3^ modified the DSEK technique using an automated microkeratome to dissect the donor lenticule (Descemet’s stripping automated endothelial keratoplasty; [DSAEK]). Later, Melles et al.^4^ described the Descemet’s membrane endothelial keratoplasty (DMEK) technique, in which the surgeon can manually prepare the donor Descemet’s membrane-endothelial layer (DE) complex.

In the 2016 Eye Banking Statistical Report of the Eye Bank Association of America, the results showed that there is an increasing trend toward DMEK surgery starting from 2011, while the use of DSAEK is gradually declining.^5^ The results also indicate that penetrating keratoplasty has shown a declining trend relative to endothelial keratoplasty in recent years. While the most common procedure for patients with Fuchs’ endothelial dystrophy (FED) and cataract was penetrating keratoplasty in the past, endothelial keratoplasty is now the most preferred technique for surgical management according to the Eye Banking Statistical Report. However, there is no consensus about the optimal management of patients with FED and cataract. Two different approaches have been described for its management: 1) the combined technique, in which the surgeon performs endothelial keratoplasty and cataract surgery in a single session, and 2) the staged technique, in which the surgeon performs the surgeries in two different sessions. Several studies have been conducted showing no difference in the final visual acuity and endothelial cell density between these two approaches.^6,7,8^

Another increasing trend in the use of DMEK surgery has been observed in patients with pseudophakic bullous keratopathy (PBK). Numerous studies have been conducted to show the efficiency of DMEK surgery in this patient group.^9^ However, the effect of different donor preparation techniques on surgical success has not been studied.

Although several studies have presented the early and late results of DMEK surgery, no results have been reported from Turkey to date. In this study, we present the initial 6-month results of patients who underwent DMEK surgery in a single tertiary center in Turkey. We share our surgical approach for patients with FED and PBK and compare the outcomes with the current literature in terms of the endothelial cell density (ECD) and best corrected visual acuity (BCVA). In addition, we evaluated the effect of different donor preparation techniques on surgical success and compared the staged and combined techniques.

## Materials and Methods

The medical records of patients who underwent DMEK for FED or PBK between 2014 and 2018 were investigated retrospectively. Patients with coexisting ocular pathology (e.g., glaucoma, uveitis) other than FED, PBK, or cataract that may interfere with BCVA and patients who had previous surgeries other than cataract surgery were excluded from the study. In addition, patients who failed to attend regular follow-ups in the first 6 months were excluded from the study (n=12). Approval from the local ethics committee was received. The study adhered to the tenets of the Declaration of Helsinki and informed consent was obtained from all patients before surgery.

BCVA, donor corneal ECD, donor age, duration in solution after obtaining the donor tissue, and duration after exitus of cadaver were evaluated preoperatively and BCVA, ECD, and ECD loss (%) at postoperative 6 months were evaluated postoperatively. Graft detachment, graft failure (development of corneal edema without any detachment), and pupillary block were recorded as surgical complications. For the patients who had cataract and FED, combined or staged procedures were performed.

ECD of the patients was evaluated with a specular microscope (Cellcheck SL Konan, Japan). Donor ECD values and other information about the donor were obtained from the Eye Bank of İstanbul University-Cerrahpaşa Cerrahpaşa Faculty of Medicine. Percentage of ECD loss was calculated as the difference between the donor ECD and ECD of the patient at postoperative 6 months. BCVA was measured using the Snellen chart, and the logarithm of the minimal angle of resolution (LogMAR) equivalent was used for statistical analysis.

Patients had complete slit-lamp examination preoperatively and at postoperative 1 day, 3-6 days, 1 month, 3 months, and 6 months, and when needed between these time points.

### DMEK Donor Graft Preparation

For dissection of the Descemet membrane graft, cornea-scleral buttons from donor globes were obtained from the cadavers and stored in Optisol GS (Bausch & Lomb Surgical, Irvine, CA, USA) solution (4°C). Endothelial cell morphology and viability were evaluated with specular microscopy in the eye bank for an optimal selection of appropriate cornea for the transplantation. Donor corneas with ECD above 2300 cells/mm^2^ were used. Donor age was between 50 and 65 years, and tissue from donors with systemic diseases that can affect graft survival was not used for the surgeries.

For DMEK graft preparation, we preferred two different approaches, one using an 8-mm donor punch for dissection and the other using a 9.5-mm donor punch.

In the first approach, after corneal trephination with the 8-mm donor punch, the endothelial side of the donor cornea was elevated with the help of a sponge and its edges were held with forceps for endothelial stripping.

**Figure 1:**
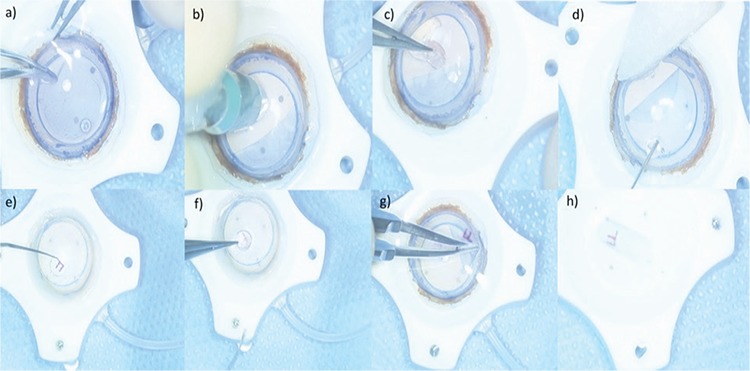
A 9.5-mm modified donor punch was used for partial corneal trephination in the donor cornea and endothelial stripping was performed with the help of a forceps (a). A corneal stromal area without the endothelial layer was obtained after stripping. Then, after a complete incision was performed with a 2-mm dermal punch over this area (b), the endothelial layer was replaced (c-d). An “F” mark was made in this area using a sterile marker and Sinskey hook (e-f). Corneal trephination was then completed with an 8-mm donor punch to yield a Descemet membrane-endothelial layer complex scroll with the “F” mark on the Descemet membrane side (g-h)

In the second approach, a 9.5-mm modified donor punch was used for partial corneal trephination in the donor cornea and endothelial stripping was performed with the help of a forceps. Firstly, a corneal stromal area without the endothelial layer was obtained after stripping. Then, after a complete incision was performed with a 2-mm dermal punch over this area, the endothelial layer was returned to its original place. Afterward, an “F” mark was made on this area with a sterile marker and a Sinskey hook. The corneal trephination was then completed with an 8-mm donor punch to yield a DE complex scroll with the “F” mark on its Descemet membrane side (Figure 1).

After corneal trephination with either of these two techniques (8 mm or 9.5 mm technique), the DE complex scroll was used for the surgery immediately after preparation. The DE complex was stored in Optisol GS corneal storage medium during preparation of the recipient bed.

### DMEK Surgical Technique

When corneal epithelial edema prevented visualization of the anterior chamber, epithelial stripping was performed for better visualization. After creating side ports with a 20-gauge (G) microvitreoretinal (MVR) blade, a circular 8-mm descemetorhexis was performed under ophthalmic viscoelastic device (OVD) with reverse Sinskey. Cohesive OVD was preferred and the OVD was removed by irrigation and aspiration after descemetorhexis. In some of the patients, peripheral iridectomy was performed with a 23-G vitrector. The donor DE complex was stained with Trypan blue.

**Figure 2:**
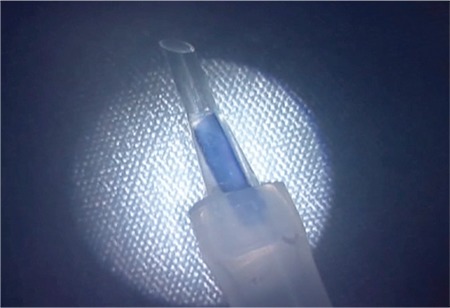
The tip of an IOL injector cartridge was combined with a silicone tubing set to create the custom-made injector

**Figure 3:**
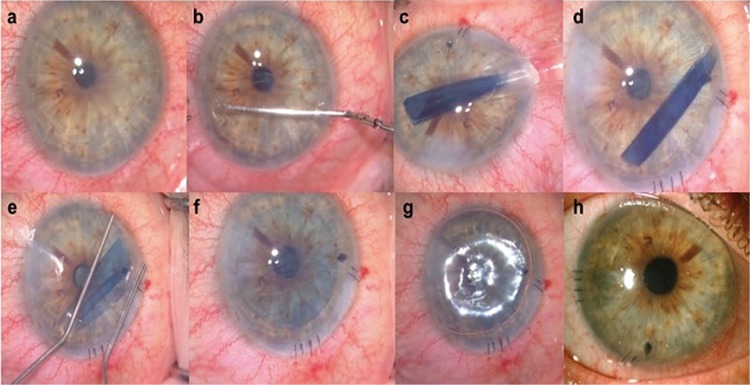
The steps of before Descemet membrane endothelial keratoplasty surgery. Preoperative slit-lamp photograph of a patient with Fuchs’ endothelial dystrophy (a). A circular 8-mm descemetorhexis was performed under ophthalmic viscoelastic device (b). The Descemet membrane-endothelial layer (DE) complex scroll was loaded by suction into the custom-made injector and the graft was injected through the corneal tunnel incision into the anterior chamber (c-d). After forming a shallow anterior chamber with the help of bimanual manipulations on the corneal surface, the donor DE complex was positioned with its endothelial side facing the iris and the DM side facing the corneal stroma (e). After complete unfolding (f), an air bubble was injected through the side port under the graft to facilitate attachment with the recipient corneal stroma (g). Slit-lamp photograph of the same patient at postoperative 2 weeks (h)

The tip of an IOL injector cartridge was combined with a silicone tubing set to create the custom-made injector (Figure 2). The DE complex scroll was loaded by suction into this injector, then injected through the corneal tunnel incision into the anterior chamber. Three 10-0 nylon sutures were used to close the main corneal incision. After forming a shallow anterior chamber with the help of bimanual manipulations on the corneal surface, the donor DE complex was placed with its endothelial side facing the iris and the Descemet membrane side facing the corneal stroma. The “F” mark was checked to ensure correct positioning of donor grafts prepared with the 9.5-mm technique. After complete unrolling, an air bubble was injected through the side port under the graft to facilitate attachment with the recipient corneal stroma. Then the anterior chamber was completely filled with air for 60-120 minutes for complete attachment and an air-fluid exchange was performed after intraocular pressure reached a level that caused the patient to feel deep pain or pressure in his/her globe. A bandage contact lens was applied on eyes that had epithelial stripping. Patients who underwent peripheral iridectomy were observed in the operating room for another hour for the development of pupillary block. The steps of the surgery are depicted in Figure 3.

### Postoperative Follow-up

Patients who underwent peripheral iridectomy were ordered to lay in supine position for 1 hour and sit for 15 minutes until 12 am. After 12 am they were ordered to lay in supine position for 2 hours and sit for 15 minutes again. In all of the patients, we observed that the remaining air filled approximately 50% on the second day and no air was observed after 3-5 days. Topical moxifloxacin and dexamethasone drops were prescribed for use every 2 hours on the first day, followed by 6 times daily. The medications were tapered until discontinuation.

### Phacoemulsification Technique (Combined and Staged)

Two different approaches were implemented for patients who had FED and cataract. The first approach was combined DMEK with phaco surgery and the second approach was staged procedure in which the patient had cataract surgery and IOL implantation firstly and had another session for DMEK.

When the combined technique was preferred, the main tunnel was kept shorter than usual. The main incision for the tunnel was 2.4 mm in all of the patients. The radius of the capsulorhexis area was 4.5-5 mm to prevent the intraocular lens (IOL) entering the anterior chamber from the capsular bag after implantation. IOL with 6 mm radius of the optic piece was used in all of the patients. Since the capsulorhexis area was small, a capsular tension ring (CTR) was used in all of the patients before IOL implantation. No dispersive viscoelastic material was used during the cataract surgery to prevent graft dislocation after DMEK. After implanting the IOL and clearing all the viscoelastic material behind the IOL, additional viscoelastic material was applied into the anterior chamber and peripheral iridectomy was performed with a 23-G vitrectomy probe. Then descemetorhexis was performed under viscoelastic material. After descemetorhexis, the main incision was enlarged to 3 mm and DMEK procedures were followed.

Epithelial scraping was performed in patients with prominent corneal edema preventing visualization of the anterior chamber before combined surgery. A bandage contact lens was applied at the end of the surgeries.

When the staged procedure was preferred, the previously described soft shell technique was utilized for the cataract surgery and DMEK was performed in another session.

### Statistical Analysis

Statistical analysis of the data was performed using SPSS software (version 21.0). The Kolmogorov–Smirnov test was used to evaluate the sample distribution. A Student’s t-test was used to compare the mean values of two independent groups with normal distribution and the Mann-Whitney U test was used to compare continuous variables with non-normal distributions. Wilcoxon’s test was used to compare the mean values of two dependent groups. P values below 0.05 were considered statistically significant.

## Results

One hundred eyes of 74 patients were included in the study. The etiology was FED in 52 eyes (52%) of 26 patients and PBK in 48 eyes (48%) of 48 patients. The mean age of the patients with FED was 67.5±5.1 years and it was 62.4±7.5 years in the patients with PBK (p=0.004). While 7 (26.9%) of 26 patients with FED were male and 19 (73.1%) were female, 28 (58.3%) of 48 patients were male and 20 (41.7%) were female in the PBK group (p=0.01). The cause of PBK was toxic anterior segment syndrome (TASS) in 4 (8.3%) of 48 eyes.

### FED vs. PBK

The baseline conditions for FED and PBK groups before DMEK procedure are summarized in Table 1. The groups were homogenous in terms of donor ECD, donor age, duration of donor tissue in solution, and duration of obtaining the donor tissue after exitus (Table 1).

When ECD loss in 6 months was compared between the two etiologies, the mean ECD of the FED eyes at 6 months after DMEK was 1719.1±152.6 cells/mm^2^ and it was 1702.2±145.9 cells/mm^2^ for the eyes with PBK (p=0.55). Mean ECD loss in 6 months was 29.2±4.4% in the FED group and 29.7±5% in the PBK group (p=0.415) (Table 2).

In patients with FED, the mean preoperative BCVA was 1.13±0.27 and it changed to 0.06±0.05 at 6 months (p<0.001). In patients with PBK, the mean preoperative BCVA was 2.36±0.69 and it changed to 0.07±0.05 at 6 months (p<0.001). There was no statistically significant difference between the groups in mean BCVA at 6 months (p=0.378) (Table 2).

### 8 vs. 9.5 mm Donor Preparation Technique

The baseline conditions before DMEK procedure for the patients who underwent different techniques (8 vs. 9.5 mm) are summarized in Table 2. The groups were homogenous in terms of donor ECD, donor age, duration of donor tissue in solution, and duration of obtaining the donor tissue after exitus (Table 3).

When ECD loss in 6 months was compared between the two graft preparation techniques, the mean ECD of the patients for whom the 8-mm technique was preferred was 1733.8±165.9 cells/mm^2^ at 6 months after DMEK and it was 1706.7±146.1 cells/mm^2^ for the patients for whom the 9.5-mm technique was preferred (p=0.356). The mean ECD loss in 6 months was 28.3±5.3% in the 8-mm group and 29.7±4.5% in the 9.5-mm group (p=0.255) (Table 4).

### Triple vs. Staged (Combined) Approach in Patients with FED and Cataract

The baseline conditions before DMEK for the patients who underwent different approaches for the management of FED and cataract (staged vs. triple) are summarized in Table 3. The groups were homogenous in terms of donor ECD, donor age, duration of donor tissue in solution, and duration of obtaining the donor tissue after exitus (Table 5).

When ECD loss in 6 months was compared between the two approaches, the mean ECD of the patients for whom the triple approach was preferred was 1738.8±166.2 cells/mm^2^ at 6 months after DMEK and it was 1702.3±140.7 cells/mm^2^ for the patients for whom the staged approach was preferred (p=0.149). The mean ECD loss in 6 months was 28.5±5.6% in the triple group and 29.8±2.9% in the staged group (p=0.279) (Table 6).

The mean preoperative BCVA was 1.09±0.28 and it changed to 0.05±0.05 at 6 months in the patients for whom the triple approach was preferred (p<0.001). The mean preoperative BCVA was 1.17±0.26 and it changed to 0.07±0.06 at 6 months in the patients for whom the staged approach was preferred (p<0.001). There was no statistically significant difference in mean BCVA at 6 months between the two groups (p=0.09) (Table 6).

### Complications

Peripheral iridectomy was not performed in 15 eyes (15%), and 3 cases of pupillary block were observed among these eyes (3%). However, no pupillary block was observed in the eyes that underwent peripheral iridectomy during surgery.

Graft failure was observed in 10 eyes (10%) and an additional DMEK surgery was performed for all of these cases. TASS was the cause of PBK in 4 of these cases. Penetrating keratoplasty was needed in 5 of these cases. Three eyes had partial graft detachment and 1 had total graft detachment, and air was applied to the anterior chamber to provide reattachment in these eyes. The patient with total graft detachment underwent re-DMEK due to suspicion of upside-down graft application. Complications of DMEK procedures in the study are summarized in Table 7.

## Discussion

In recent years, endothelial keratoplasty techniques (DMEK and DSAEK) have been the major surgical approach for the management of FED and PBK. Although penetrating keratoplasty is still in use, it has the disadvantages of complications, lower patient satisfaction, and lower BCVA. However, endothelial keratoplasty techniques, especially DMEK, require more surgical experience. Despite this drawback, after enough surgeries, it can be performed in any center because special surgical equipment is not necessary for this surgical approach, unlike DSAEK. In DMEK, the surgeon has the advantage of preferring the best approach for the patient in each step of donor tissue preparation. Furthermore, in a recent meta-analysis, DMEK was found to show better postoperative results regarding BCVA, patient satisfaction, and graft-related issues.^10^ In this study, we presented our results of the increasingly popular DMEK surgery in 100 eyes with FED or PBK.

In clinical studies, the success of DMEK surgery is usually evaluated based on both ECD loss and change in BCVA. While Droutsas et al.^11^ showed 31.6% ECD loss at 6 months after DMEK surgery for the treatment of patients with FED, Ham et al.^12^ showed 28.4% ECD loss. Consistent with these previous studies, we observed mean ECD loss at 6 months of 29.2±4.4% in the FED group and 29.7±5% in the PBK group. Our study also showed that there was no difference between the FED and PBK patients in terms of ECD loss at 6 months. This indicates that DMEK surgery might be equally successful in terms of ECD in patients with FED and PBK.

In general practice, the 9.5 mm technique is preferred for donor graft preparation.^13^ In our study, we evaluated whether there is a difference between the 9.5 mm and 8 mm techniques. Although contact with the endothelial layer during the 8 mm preparation technique might cause concern about increased ECD loss, we did not observe any significant increase in loss. Our results showed that both techniques can be used effectively with comparable endothelial cell loss.

Although penetrating keratoplasty was the main approach in the past, recent advances in endothelial keratoplasty techniques have made it the main approach for patients with FED. However, there is controversy regarding the best approach to patients with FED and cataract. This issue is important because the rate of cataract formation within 1 year after any endothelial keratoplasty was reported to be as high as 40%.^14^ Two different approaches have been described in the literature. In the combined technique, the surgeon can perform the DMEK surgery together with phacoemulsification in the same session, whereas in the staged technique, DMEK is performed in another session after phacoemulsification. Previous studies offered conflicting results about the success of both approaches. Most of the studies suggested that the two techniques were similar in terms of final BCVA and ECD.^6,7^ Schoenberg et al.^8^ reported the results of 108 triple DMEK procedures and found that triple DMEK safely achieved excellent BCVA. Sykakis et al.^6^ reported increased graft dislocation rate in the combined technique. However, this increase was attributed to the use of Healon-GV rather than Healon. Similar to the previous studies, we did not observe any difference between the techniques in terms of ECD loss or BCVA at 6 months in our study.

Graft failure is one of the complications of DMEK surgery. Re-DMEK, back-up DSEK, or penetrating keratoplasty can be used for the management of graft failure.^15^ Heinzelmann et al.^16^ showed that pre-cut donor graft was linked to increased graft failure rate. Thus, donor tissue preparation should be performed immediately before surgery. In our study, graft failure was observed in 10 eyes (10%) and an additional DMEK procedure was performed for all of these cases. TASS was the cause of PBK in 4 of these cases. Penetrating keratoplasty was needed in 5 of these cases. Although previously we showed that DSAEK was successful in cases of chronic TASS in terms of visual and anatomical outcomes,^17^ our study suggests that DMEK might not be a good approach for patients with PBK secondary to TASS. However, further studies with a larger number of patients should be conducted to compare the success of DMEK and DSAEK for the treatment of PBK secondary to TASS.

Another complication of the DMEK surgery is graft detachment. This complication can be managed with re-bubbling. Although the rates of total and partial graft detachment were 30% and 62-63% in initial reports,^18,19,20,21,22^ the detachment rate decreased to as low as 4-34.6% in recent years due to increased surgical experience.^19^ In our study, 3 eyes (3%) with partial graft detachment and 1 eye (1%) with total graft detachment were observed and air was applied to the anterior chamber to provide reattachment. Suspecting upside down graft application, we performed re-DMEK in the patient with total graft detachment.

### Study Limitations

The relatively short follow-up time, small number of patients with PBK secondary to TASS, and the retrospective, non-randomized, and descriptive design of the study are limitations of our study. Due to the non-randomized and descriptive nature of the study, some of our findings may lack generalizability. In addition, central corneal thickness data were not included in the study.

## Conclusion

DMEK was found to be a safe and effective method for patients with FED and PBK. In patients with FED together with cataract, we did not observe any difference in final BCVA or ECD between the staged or combined procedures, which indicates that both approaches can be used efficiently in these patients. Furthermore, no difference in 6-month ECD was found between graft preparation using the 8 mm or 9.5 mm techniques. Further studies including central corneal thickness data should be performed to investigate the results of the increasingly popular DMEK procedure.

## Figures and Tables

**Table 1:**
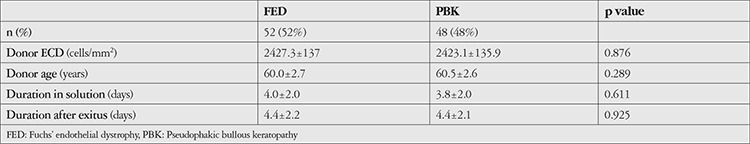
Comparison of baseline conditions before Descemet membrane endothelial keratoplasty surgery

**Table 2:**
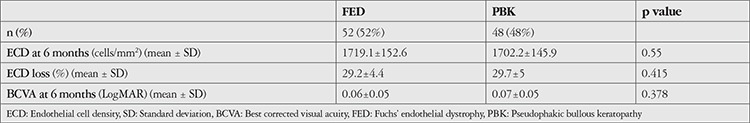
Endothelial cell density (ECD) at 6 months, ECD loss in 6 months, and best corrected visual acuity at 6 months in patients with Fuchs’ endothelial dystrophy and pseudophakic bullous keratopathy

**Table 3:**
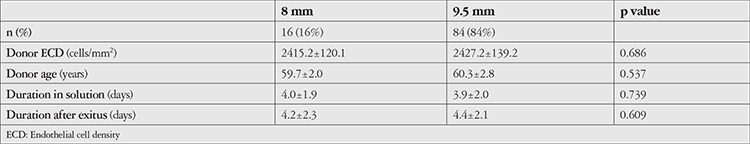
Comparison of baseline conditions according to different graft techniques before Descemet membrane endothelial keratoplasty surgery

**Table 4:**
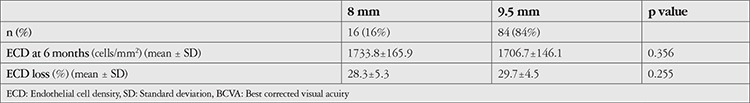
Endothelial cell density (ECD) at 6 months and ECD loss in 6 months according to the different graft preparation techniques

**Table 5:**
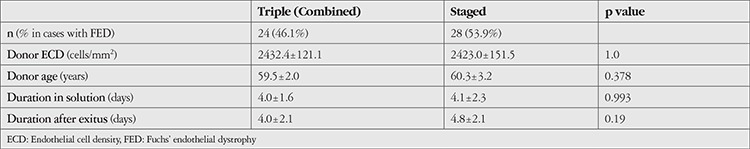
Comparison of baseline conditions according to the different approaches used in the management of cataract

**Table 6:**
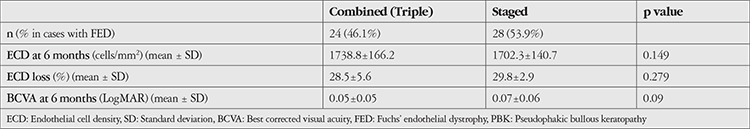
Endothelial cell density (ECD) at 6 months, ECD loss in 6 months, and best corrected visual acuity at 6 months according to the different approaches for patients with cataract and Fuchs’ endothelial dystrophy

**Table 7:**
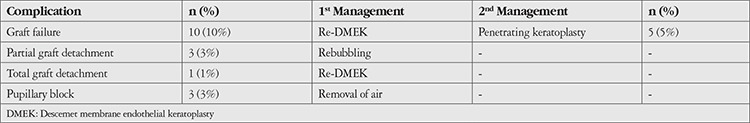
Complications of before Descemet membrane endothelial keratoplasty and their management

## References

[1] Terry MA, Ousley PJ (2001). Deep lamellar endothelial keratoplasty in the first United States patients: early clinical results. Cornea..

[2] Melles GR, Wijdh RH, Nieuwendaal CP (2004). A technique to excise the descemet membrane from a recipient cornea (descemetorhexis). Cornea.

[3] Gorovoy MS (2006). Descemet-Stripping Automated Endothelial Keratoplasty. Cornea..

[4] Melles GR, Ong TS, Ververs B, van der Wees J (2006). Descemet Membrane Endothelial Keratoplasty (DMEK). Cornea..

[5] America EBA of (2017.). 2016 Eye Banking Statistical Report. Eye Bank Assoc Am..

[6] Sykakis E, Lam FC, Georgoudis P, Hamada S, Lake D (2015). Patients with Fuchs Endothelial Dystrophy and Cataract Undergoing Descemet Stripping Automated Endothelial Keratoplasty and Phacoemulsification with Intraocular Lens Implant: Staged versus Combined Procedure Outcomes. J Ophthalmol..

[7] Laaser K, Bachmann BO, Horn FK, Cursiefen C, Kruse FE (2012). Descemet Membrane Endothelial Keratoplasty Combined With Phacoemulsification and Intraocular Lens Implantation: Advanced Triple Procedure. Am J Ophthalmol..

[8] Schoenberg ED, Price FW Jr, Miller J, McKee Y, Price MO (2015). Refractive outcomes of Descemet membrane endothelial keratoplasty triple procedures (combined with cataract surgery). J Cataract Refract Surg..

[9] Liarakos VS, Ham L, Dapena I, Tong CM, Quilendrino R, Yeh RY, Melles GR (2013). Endothelial keratoplasty for bullous keratopathy in eyes with an anterior chamber intraocular lens. J Cataract Refract Surg..

[10] Marques RE, Guerra PS, Sousa DC, Gonçalves AI, Quintas AM, Rodrigues W (2019). DMEK versus DSAEK for Fuchs’ endothelial dystrophy: A meta-analysis. Eur J Ophthalmol..

[11] Droutsas K, Ham L, Dapena I, Geerling G, Oellerich S, Melles G (2010). [Visual acuity following Descemet-membrane endothelial keratoplasty (DMEK): first 100 cases operated on for Fuchs endothelial dystrophy]. Klin Monbl Augenheilkd..

[12] Ham L, Dapena I, van Luijk C, van der Wees J, Melles GR (2009). Descemet membrane endothelial keratoplasty (DMEK) for Fuchs endothelial dystrophy: review of the first 50 consecutive cases. Eye..

[13] Tausif HN, Johnson L, Titus M, Mavin K, Chandrasekaran N, Woodward MA, Shtein RM, Mian SI (2014). Corneal donor tissue preparation for Descemet’s membrane endothelial keratoplasty. J Vis Exp..

[14] Tsui JY, Goins KM, Sutphin JE, Wagoner MD (2011). Phakic Descemet Stripping Automated Endothelial Keratoplasty: Prevalence and Prognostic Impact of Postoperative Cataracts. Cornea..

[15] Dapena I, Ham L, van Luijk C, van der Wees J, Melles GR (2010). Back-up procedure for graft failure in Descemet membrane endothelial keratoplasty (DMEK). Br J Ophthalmol..

[16] Heinzelmann S, Böhringer D, Eberwein P, Reinhard T, Maier P (2017). Graft dislocation and graft failure following Descemet membrane endothelial keratoplasty (DMEK) using precut tissue: a retrospective cohort study. Graefe’s Arch Clin Exp Ophthalmol..

[17] Arslan OS, Ünal M, Arici C, Görgün E, Yenerel M, Cicik E (2010). Descemetstripping automated endothelial keratoplasty in eyes with toxic anterior segment syndrome after cataract surgery. J Cataract Refract Surg..

[18] Melles GR, Ong TS, Ververs B, van der Wees J (2008). Preliminary Clinical Results of Descemet Membrane Endothelial Keratoplasty. Am J Ophthalmol..

[19] Ang M, Wilkins MR, Mehta JS, Tan D (2016). Descemet membrane endothelial keratoplasty. Br J Ophthalmol..

[20] Guerra FP, Anshu A, Price MO, Giebel AW, Price FW (2011). Descemet’s Membrane Endothelial Keratoplasty. Ophthalmology..

[21] Guerra FP, Anshu A, Price MO, Price FW (2011). Endothelial Keratoplasty: Fellow Eyes Comparison of Descemet Stripping Automated Endothelial Keratoplasty and Descemet Membrane Endothelial Keratoplasty. Cornea..

[22] Price MO, Giebel AW, Fairchild KM, Price FW Jr (2009). Descemet’s membrane endothelial keratoplasty: prospective multicenter study of visual and refractive outcomes and endothelial survival. Ophthalmology..

